# Lipopolysaccharide and palmitic acid synergistically induced MCP-1 production via MAPK-meditated TLR4 signaling pathway in RAW264.7 cells

**DOI:** 10.1186/s12944-019-1017-4

**Published:** 2019-03-25

**Authors:** Xuehong Wang, Xin Jiang, Bin Deng, Juan Xiao, Junfei Jin, Zhaoquan Huang

**Affiliations:** 1 Department of Pathology, Xiangya Hospital, Central South University, Changsha, 410008 Hunan China; 2 Department of Pathology, the Affiliated Hospital of Guilin Medical University, 15 Lequn Road, Guilin, 541001 Guangxi China; 3 Laboratory of Hepatobiliary and Pancreatic Surgery, Affiliated Hospital of Guilin Medical University, 15 Lequn Road, Guilin, 541001 Guangxi China; 4 China-USA Lipids in Health and Disease Research Center, Guilin Medical University, Guilin, 541001 Guangxi China; 5 Guangxi Key Laboratory of Molecular Medicine in Liver Injury and Repair, Guilin, 541001 Guangxi China

**Keywords:** Palmitic acid, Lipopolysaccharide, MCP-1, MAPK, TLR4

## Abstract

**Background:**

Obesity increases the risk of developing diabetes mellitus. Clinical studies suggest that risk factors like palmitic acid (PA) and lipopolysaccharide (LPS) exist simultaneously in diabetes with obesity. Combination of PA and LPS even at low concentration can induce strong inflammatory reaction. Monocyte chemoattractant protein-1 (MCP-1) is an important inflammatory chemokine related to insulin resistance and type II diabetes. Our previous study using PCR array revealed that LPS and PA synergistically induce MCP-1 mRNA expression in macrophage cells RAW264.7, while the protein expression of MCP-1 in this case was not investigated. Moreover, the underling mechanism in the synergistic effect of MCP-1 expression or production induced by treatment of LPS and PA combination remains unclear.

**Methods:**

Protein secretion of MCP-1 was measured by the enzyme-linked immunosorbent assay (ELISA) and mRNA levels of MCP-1 and Toll-like receptor 4 (TLR4) were measured by real-time PCR. Statistical analysis was conducted using SPSS software.

**Results:**

LPS could increase MCP-1 transcription as well as secretion in RAW264.7, and PA amplified this effect obviously. Meanwhile, combination of LPS with PA increased TLR4 mRNA expression while LPS alone or PA alone could not, TLR4 knockdown inhibited MCP-1 transcription/secretion induced by LPS plus PA. Moreover, not NF-κB inhibitor but inhibitors of mitogen-activated protein kinase (MAPK) signaling pathways, including c-Jun NH2-terminal kinase (JNK), extracellular signal-regulated kinase (ERK), and p38 MAPK were found to block MCP-1 generation stimulated by LPS plus PA.

**Conclusion:**

LPS and PA synergistically induced MCP-1 secretion in RAW264.7 macrophage cells, in which MCP-1 transcription mediated by MAPK/TLR4 signaling pathways was involved. Combined treatment of PA and LPS in RAW264.7 cells mimics the situation of diabetes with obesity that has higher level of PA and LPS, MAPK/TLR4/ MCP-1 might be potential therapeutic targets for diabetes with obesity.

## Introduction

As a chronic metabolic disease, diabetes mellitus as well as its complications impose a great economic burden on individuals worldwide, due to the frequency of diagnosed cases and consequential increases in medical costs [1]. However, the mechanism underlying diabetes has not been fully elucidated by now. Recently, low-grade chronic inflammation which was meditated by cytokines and chemokines has been found to be important in diabetes [2, 3]. Inflammatory mediators are considered to be predictors for obesity and diabetes [4]. Specifically, the serum level of monocyte chemoattractant protein-1 (MCP-1) is significantly increased in mice or patients with type II diabetes [5, 6], and MCP-1 is reported to be a major contributor to the inflammatory process associated with diabetes [7]. Meanwhile, MCP-1 has been demonstrated as an important risk factor during the development of insulin resistance and type II diabetes [4]. Besides, serum levels of MCP-1 are positively correlated with tumor stage and grade in tumors including pancreatic cancer [5–9]. However the roles of MCP-1 in various diseases especially in diabetes are still not fully understood.

Diabetes incidence increases in people with obesity characterized as higher content of saturated fatty acid (FA). Data from type II diabetes patients with obesity shows that FA is correlated to chronic low-grade inflammation or insulin resistance [10]. Moreover, increased palmitic acid (PA), an abundant FA in plasma, contributes to dyslipidemia in type II diabetes [11], so PA is an important FA involved in diabetes. Many clinical studies report that blood circulating lipopolysaccharide (LPS) is increased in patients with obesity [12]. LPS, which is the major component of the cell wall of Gram-negative bacteria, triggers inflammation response and activates tissue inflammatory factors through TLR4 signaling pathway [13]. Increased FA (especially PA) and elevated LPS may exist simultaneously in type II diabetes with obesity. However, how PA and LPS work in the development of type II diabetes is not fully understood.

Our previous study showed that PA could amplify the inflammation response by LPS-stimulated in macrophage cell line RAW264.7, PCR array screening revealed that PA and LPS synergistically increase MCP-1 mRNA expression [14]. However, whether PA could augment MCP-1 protein production induced by LPS-stimulated was not investigated, and the underlying mechanism of this synergistic effect on MCP-1 was unknown.

In this study, the effect of LPS combined with PA on MCP-1 protein generation was investigated in RAW264.7 cells. The results suggested that the underlying mechanism of synergistic generation of MCP-1 induced by PA plus LPS was related to MCP-1 transcription and MAPK-meditated TLR4 signaling pathways.

## Methods

### Chemical and reagent

The PA, LPS from *E. coli* and actinomycin D were purchased from Sigma-Aldrich (St. Louis, MO). MCP-1 Elisa kit was from R&D Systems manufacturer (Minneapolis, MN) PD-98059, SP-600125, Bay-117,085 were obtained from Selleck Chemicals (Houston, TX). SB-203580 was from Merck Millipore (Billerica, MA). TRIzol reagent was purchased from Life Technologies (Gaithersburg, MD) and SYBR Premix were from TaKaRa (Tokyo, Japan). FastQuant RT Kit (with gDNase) was obtained from TIANGEN (Beijing, China). TLR4 or control siRNA was purchased from Genepharma (Shanghai, China) and Lipofectamine 2000 was purchased from Life Technologies (Gaithersburg, MD). DMEM and fetal bovine serum (FBS) were obtained from Thermo Scientific (Rockford, IL).

### Cells and treatment

RAW264.7 cells, purchased from American Type Culture Collection (Manassas, VA) were cultured in DMEM containing 10% heat-inactivated FBS. The cells were cultured in a humidified atmosphere of 5% CO_2_ at 37 °C. For compound treatment, LPS from *E. coli* and PA were used. RAW264.7 cells were treated with 1 ng/ml LPS, 100 μM PA, or 1 ng/ml LPS together with 100 μM PA for 24 h. After that, culture medium was subjected to ELISA assay for MCP-1 secretion, and cells were collected for quantification of mRNA analysis.

### PA preparation

PA was dissolved in 0.1 N NaOH and 70% ethanol at 70 °C at 200 mM. The solution was kept at 55 °C for 10 min, then mixed, and cooled down to room temperature.

### Cytotoxicity assay

RAW264.7 cells (5 × 10^3^/well) were seeded into 96-well plates and cultured for 24 h, then the cells were treated with 100 μM PA, 1 ng/ml LPS, or 1 ng/ml LPS together with 100 μM PA for 24 h, finally cytotoxicity was assessed using the Cell Counting Kit-8 (CCK-8; DOJINDO, Shanghai, China). The CCK-8 reagent was added to cells at 37 °C for 1 h, and the optical density was measured by a microplate reader setting at 450 nm.

### Enzyme-linked immunosorbent assay

MCP-1 in medium was quantified using sandwich enzyme-linked immunosorbent assay (ELISA) kits according to the protocol.

### Real-time PCR

Total RNA was isolated from cells using the TRIzol reagent. First-strand complementary DNA (cDNA) was synthesized from 1 μg of total RNA using FastQuant RT Kit (with gDNase). The flowering primers were used: mouse MCP-1 forward, 5′-GCAGGTCCCTGTCATGCTTC-3′, reverse, 5′-ACAGCTTCTTTGGGACACCT-3′; mouse CCR2 forward, 5′-ACAGCTCAGG ATTAACAGGGACTTG-3′, reverse, 5′-ACCACTTGCATGCACACATGAC-3′; mouse GADPH forward, 5′-CACCATCTTCCAGGAGCGAG-3′, reverse, 5′-GACTCCACGACGTACTCAGC-3′; mouse TLR4 forward, CTTCCACAAGAGCGGAAGG, reverse, CAGCAGGGACTTCTCAACCT. Real-time PCR was performed in duplicate three times using 8 μl of the reaction mixture containing 1.0 μl of cDNA, 0.1 μM each primer, and 4 μl of SYBR Premix. The relative level of gene expression was quantified using the comparative CT method, normalized to the GAPDH, and expressed as the fold induction of the control.

### MCP-1 mRNA stability analysis

RAW264.7 cells were plated into 6-well plates (0.5 × 10^6^ cells) and treated with 1 ng/ml LPS, or 100 μM PA combined with 1 ng/ml LPS for 12 h, in the presence or absence of actinomycin D (10 μg/ml) for pretreatment 2 h. Then RAW264.7 cells were harvested and MCP-1 mRNA was quantified using real-time PCR as described above.

### RNA interference

RAW264.7 cells were transiently transfected with 200 pmol TLR4 siRNA or the negative control siRNA using Lipofectamine 2000 according to the manufacturer’s instructions. Twelve hours later, transfected cells were treated with 1 ng/ml LPS, 100 μM PA, or LPS plus PA for additional 24 h.

### Statistical analysis

Data are presented as means±SD. Analyses were performed using SPSS 19.0 software (SPSS, Inc., USA). One-way analysis of variance (ANOVA), two-way repeated measures ANOVA and student’s t test were applied to determine the statistical significance among different experimental groups. *P* < 0.05 was considered statistically significant.

## Results

### PA amplified MCP-1 secretion in RAW264.7 macrophage triggered by low-concentration of LPS

**Fig. 1:**
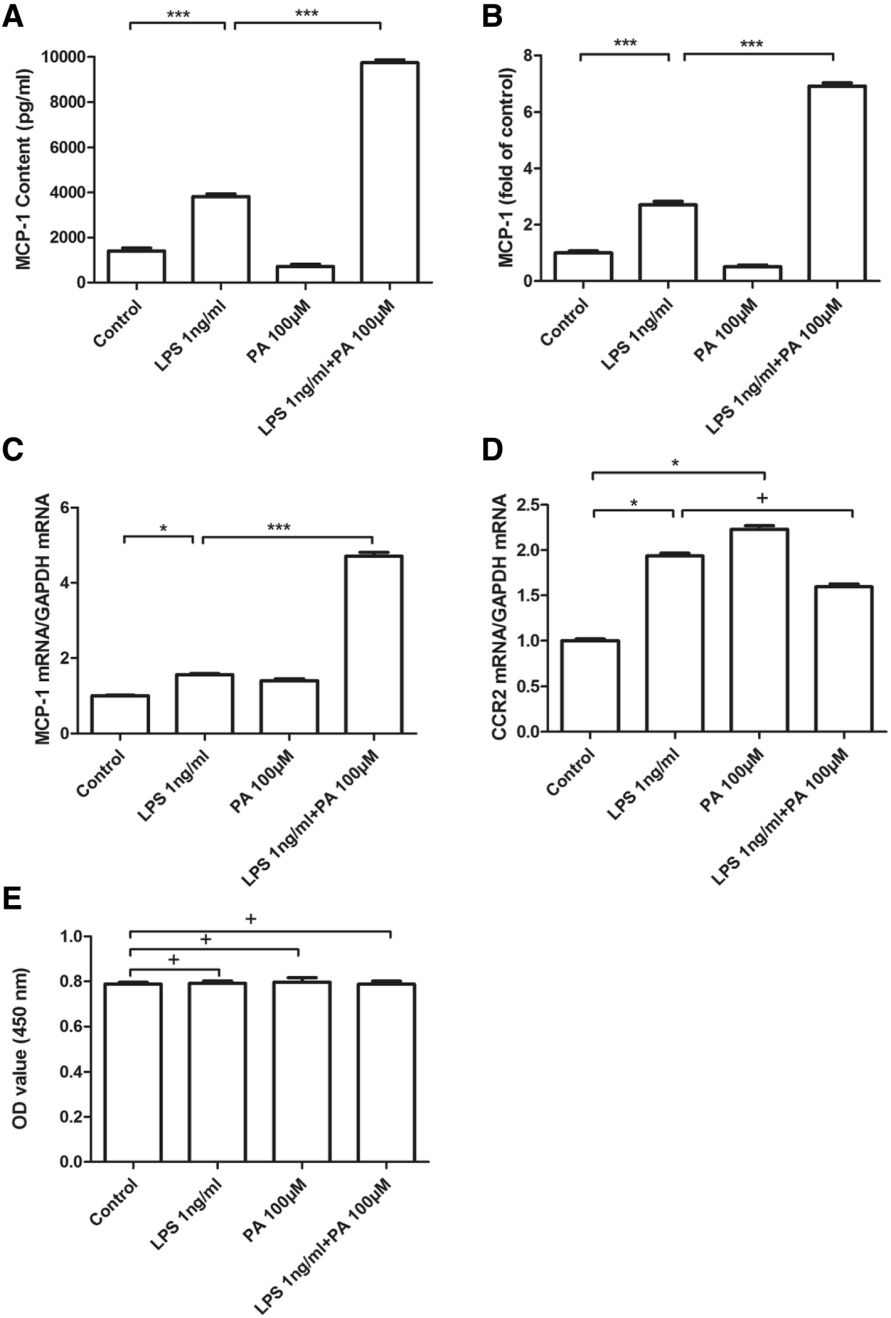
The markedly synergistic effect of PA on LPS-stimulated MCP-1 secretion from RAW264.7. RAW264.7 cells were treated with LPS 1 ng/ml, in the absence or presence of PA 100 μM for 24 h. After treatment, MCP-1 secretion in the culture medium were measured by ELISA, and MCP-1 was showed as pg/mL (**a**) or fold of control (**b**). Cells were collected and subject to MCP-1 (**c**) and CCR2 (**d**) mRNA analysis by real-time PCR, or cell survival was assayed by CCK8 kit (**e**). ^***^ *P <* 0.01; ^*^ 0.01 < *P <* 0.05; ^+^ *P >* 0.05. The data from three independent experiments were represented as mean ± SD

In order to investigate the synergistic effect on MCP-1 generation mediated by LPS and PA, the secreted protein of MCP-1 in the medium and the MCP-1 mRNA expression in cells were measured. The secretion of MCP-1 was stimulated by LPS alone, but not by PA alone; interestingly, PA augmented LPS-mediated MCP-1 generation (Fig. 1a and b). Similar results of MCP-1 mRNA in RAW264.7 cells under treatment of LPS alone or LPS plus PA were observed (Fig. 1c). Besides, the mRNA level of CCR2 which is the receptor of MCP-1 was induced by LPS alone and PA alone, but no synergistic increase on CCR2 mRNA appeared under treatment of LPS combined with PA (Fig. 1d). Notably, 100 μM PA, 1 ng/ml LPS, or 1 ng/ml LPS together with 100 μM PA did not affect cell survival indicating there are no cytotoxicity (Fig. 1e). These data suggested that LPS could stimulate MCP-1 production, the synergistic increase was obvious in case of LPS coupled with PA treatment, but CCR2 was not involved in this synergy.

### PA increased LPS-stimulated MCP-1 expression by enhancing MCP-1 transcription

**Fig. 2:**
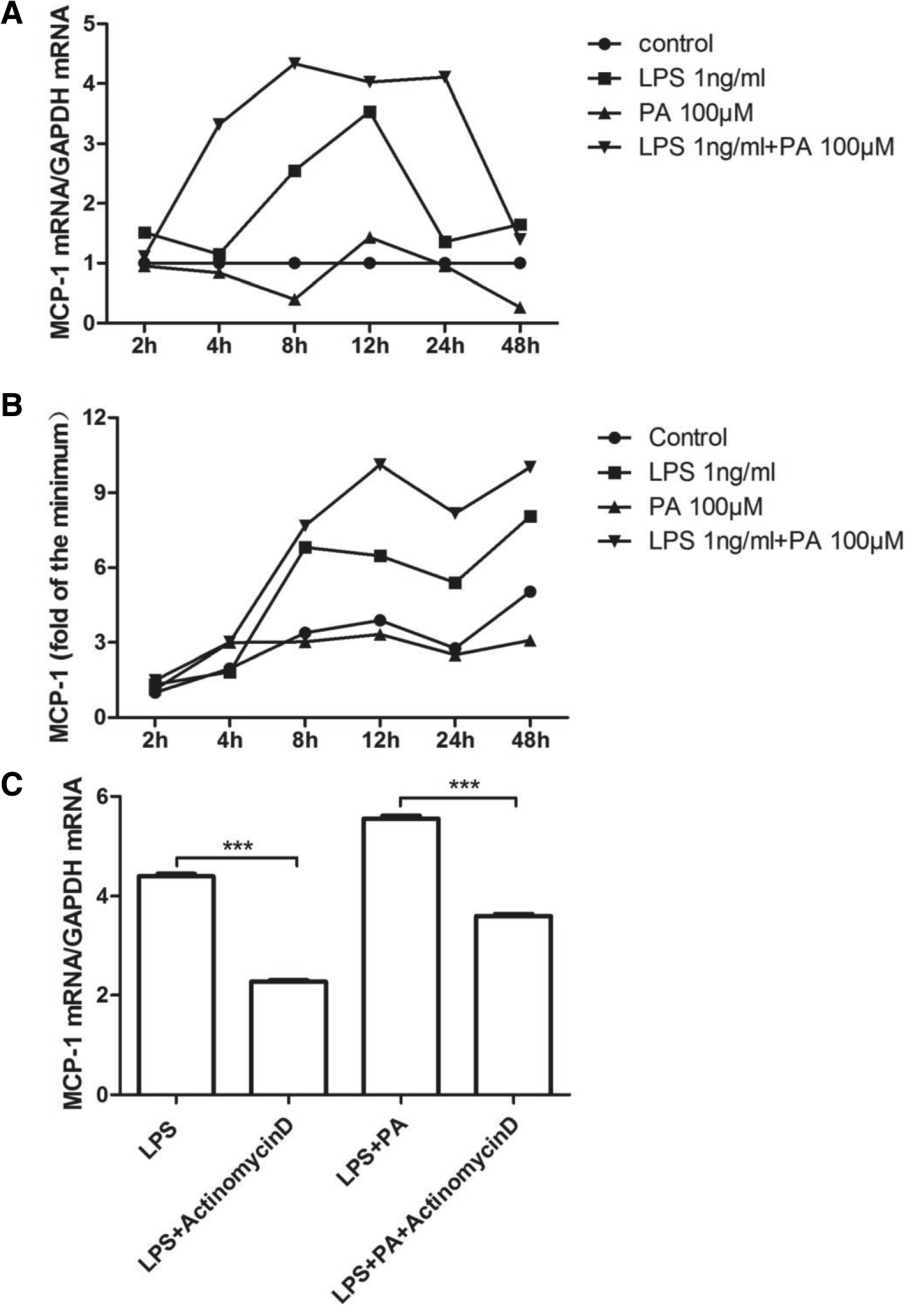
Time course of MCP-1 secretion and mRNA expression by RAW264.7 cells treated with LPS, PA, or LPS plus PA. RAW264.7 cells were treated with 1 ng/ml of LPS, 100 μM of PA, or LPS plus PA for different times as indicated. At each time point, culture medium was collected and RNA isolated from cells. MCP-1 in culture medium (**a**) and MCP-1 mRNA in cells (**b**) were quantified using ELISA and real-time PCR, respectively. The data from three independent experiments are represented as mean ± SD, and the MCP-1 secretion was showed by the fold of control. Two-way repeated measures ANOVA were applied to determine the statistical significance. **c** The effect of LPS or the combination of LPS and PA on MCP-1 mRNA transcription. RAW264.7 cells were treated with 1 ng/ml LPS or 1 ng/ml LPS plus 100 μM PA for 12 h, followed by addition of 10 μg/ml actinomycin D The cells were harvested after the addition of actinomycin D for 2 h, and MCP-1 mRNA was quantified using real-time PCR. Student's t test were used. ^***^ *P <* 0.01. The data were represented as mean ± SD from three independent experiments

Because under PA plus LPS treatment the synergistic increase appeared in both mRNA and protein of MCP-1, transcription regulation of MCP-1 might be involved in this synergy. To confirm this hypothesis, the kinetics of both MCP-1 protein secretion and MCP-1 mRNA expression were investigated. As Fig. 2 showed, PA nearly had no effect on MCP-1 mRNA expression and its secretion. Under LPS treatment, the level of MCP-1 mRNA was increased, peaked at 12 h, and then decreased gradually (Fig. 2a). Under LPS plus PA treatment, the level of MCP-1 mRNA was increased before 8 h, plateaued from 8 h to 24 h, and then decreased gradually (Fig. 2a). Statistical analysis showed that LPS alone, LPS plus PA significantly increased MCP-1 mRNA expression, but LPS plus PA showed more impact on MCP-1 mRNA expression than LPS alone (except for 48 h). Interestingly, the trends of increase in MCP-1 secretion looks similar between LPS and LPS plus PA, MCP-1 secretion peaked at 8 h under LPS treatment, and it peaked at 12 h exposed to LPS plus PA (Fig. 2b). Similarly, LPS plus PA induced more MCP-1 secretion than LPS or PA alone. In addition, synergetic effect by LPS plus PA appeared in both mRNA and protein of MCP-1 (Fig. 2a and b). Moreover, transcription inhibitor actinomycin D reduced the elevation of MCP-1 mRNA induced by LPS plus PA, indicating MCP-1 transcription not mRNA stability was involved in PA increased LPS-stimulated MCP-1 expression.

### PA amplified LPS–stimulated MCP-1 generation via TLR4 signaling

**Fig. 3:**
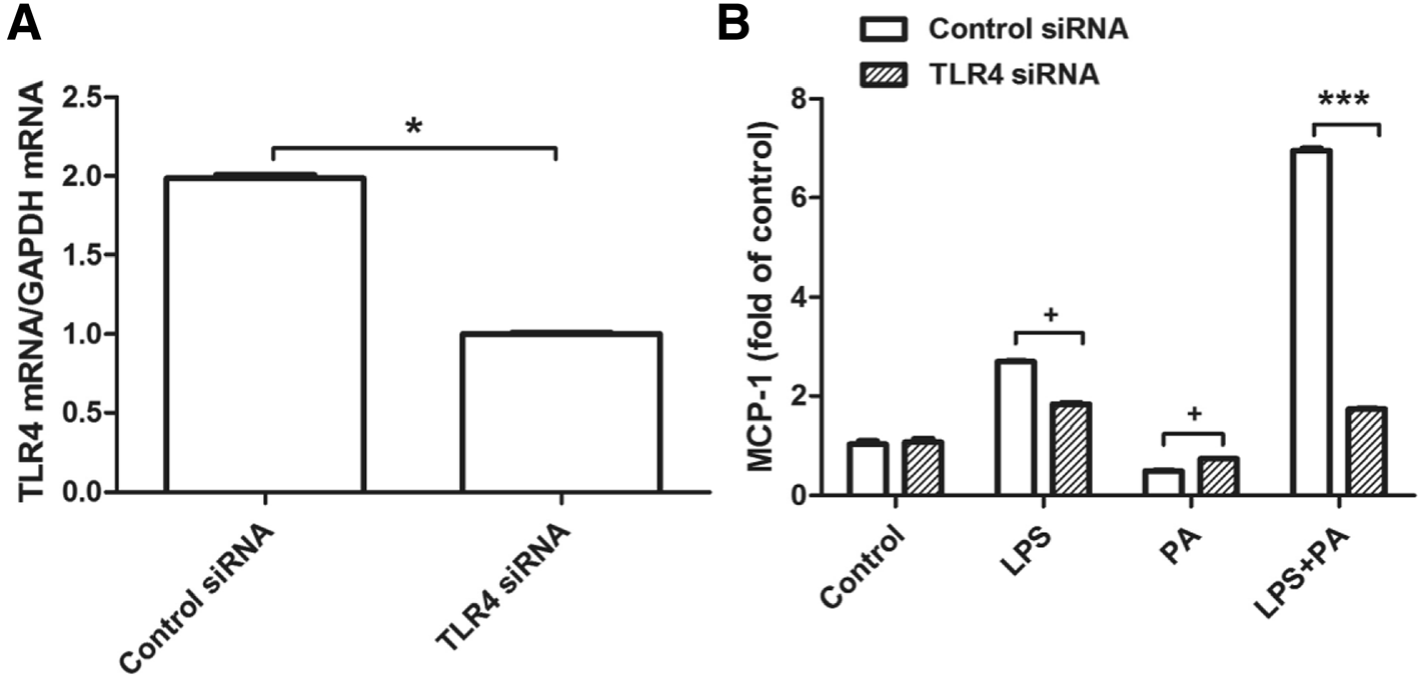
RAW264.7 cells were transfected with 200 pmol TLR4 siRNA or negative control siRNA for 12 h, then cells were treated with 1 ng/ml LPS, 100 μM PA, or LPS plus PA for 24 h, finally MCP-1 secretion was quantified using ELISA. TLR4 knockdown by siRNA was confirmed using real-time PCR (**a**). MCP-1 secretion was showed by the fold of control (**b**). ^***^ *P <* 0.01; ^*^ 0.01 < *P <* 0.05; ^+^ *P >* 0.05. The data were represented as mean ± SD from three independent experiments

It had been known that LPS specifically binds to TLR4 and activates its downstream signaling pathway, causing inflammation characterized as inflammatory factor upregulation [13]. Thus the role of TLR4 on the MCP-1 secretion induced by LPS combined with PA was investigated. As we expect, TLR4 knockdown in RAW264.7 cells largely inhibited MCP-1 secretion triggered by LPS or LPS plus PA (Fig. 3). Of note, in cells treated with LPS plus PA, TLR4 knockdown decreased MCP-1 secretion to the similar level in LPS treated cells (Fig. 3). Surprisingly, TLR4 knockdown increased MCP-1 secretion slightly under PA treatment. Therefore, these data showed that PA amplified the effect of LPS-induced MCP-1 generation through TLR4.

### The MAPK pathway was involved in MCP-1 marked increase by PA plus LPS

**Fig. 4:**
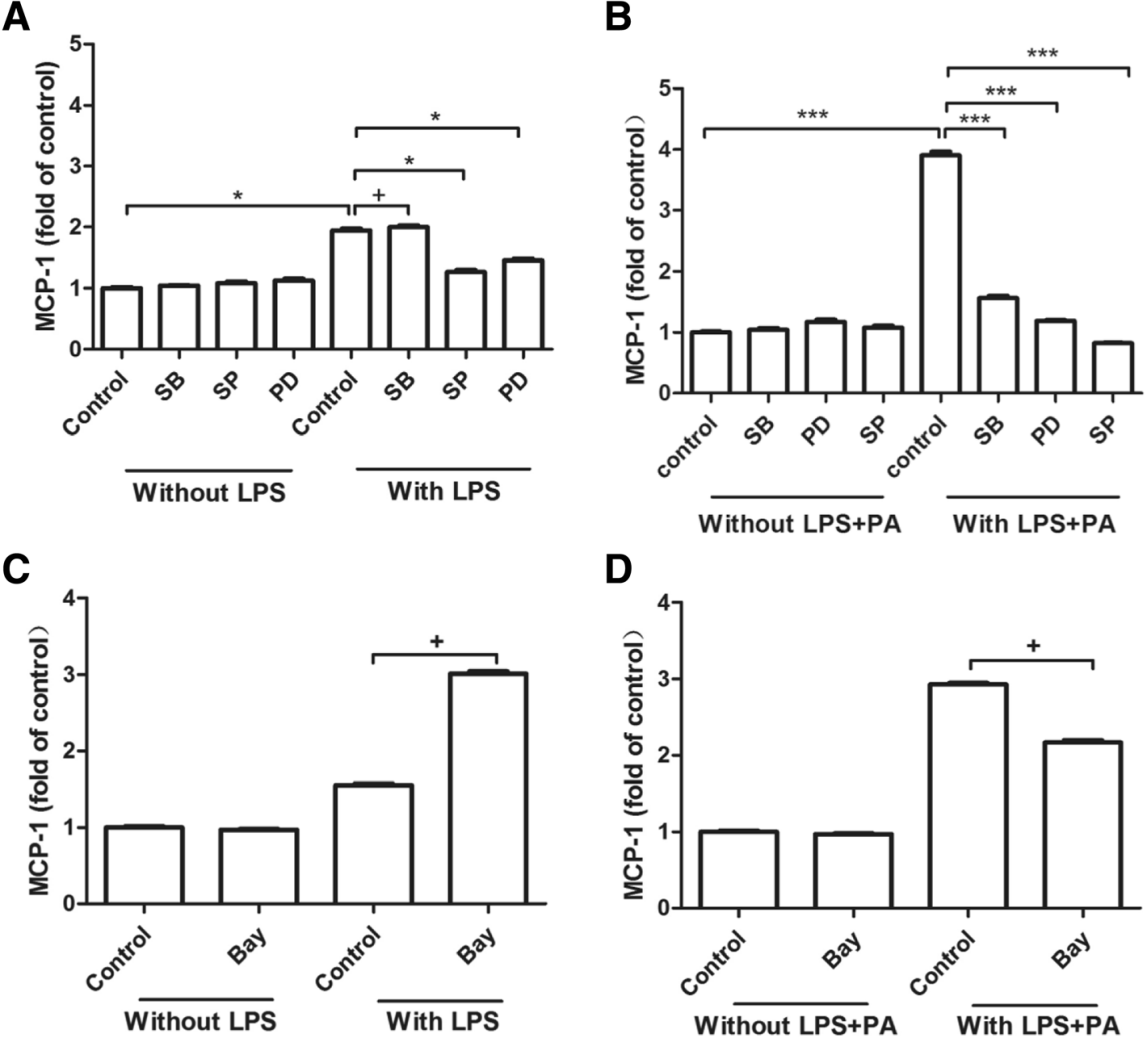
The effect of pharmacological inhibitors of MAPK and NF-κB signaling pathways on MCP-1 secretion stimulated by LPS alone or the combination of LPS and PA. A-B: RAW264.7 cells were treated with 1 ng/ml LPS alone (**a**) or 1 ng/ml LPS + 100 μM PA (**b**) in the absence or presence of 10 μM SB-203580 (SB), an inhibitor for the p38 MAPK pathway, 2.5 μM SP-600125 (SP), an inhibitor for the JNK pathway, or 2.5 μM PD 98059 (PD), an inhibitor for the ERK pathway, for 24 h. C-D: RAW264.7 cells were also treated with 1 ng/ml LPS alone (**c**) and the combination of 1 ng/ml LPS + 100 μM PA (**d**) in the absence or presence of 0.5 μM Bay-117,085 (Bay), an inhibitor for the NF-κB pathway, for 24 h. After treatment, MCP-1 in culture medium was quantified using ELISA. The RAW264.7 were pretreated with all the inhibitors for 30 min, following LPS or LPS plus PA treatment for 24 h. MCP-1 secretion was showed by the fold of control, and the data from three experiments were presented. ^***^ *P <* 0.01; ^*^ 0.01 < *P <* 0.05; ^+^ *P >* 0.05

MAPK pathway and NF-κB pathway were involved in LPS-inhibited MCP-1 generation in cultured astrocytes or LPS plus PA-stimulated IL-6 secretion in RAW264.7 [14, 15]. Therefore, LPS or LPS plus PA induced MCP-1 generation in RAW264.7 cells might be related to abovementioned pathways, pharmacological inhibitors of MAPK and NF-κB pathways were used to validate this hypothesis. Our results showed that MCP-1 secretion induced by LPS combined with PA was inhibited significantly by JNK inhibitor SP-600125 (SP), ERK inhibitor PD-98059 (PD) or p38 inhibitor SB-203580 (SB) (Fig. 4b). However, MCP-1 secretion induced by LPS alone was inhibited by SP and PD but not SB (Fig. 4a). In addition, MCP-1 secretion induced by LPS alone was not affected by NF-κB inhibitor Bay117082, which also showed similar effect in RAW264.7 cells treated with LPS plus PA treatment (Fig. 4c and d). Therefore, the MAPK pathway was probably involved in MCP-1 upregulation induced by PA combined with LPS, but the NF-κB pathway was not.

### MAPK regulated TLR4 affecting MCP-1 generation mediated by LPS plus PA

**Fig. 5:**
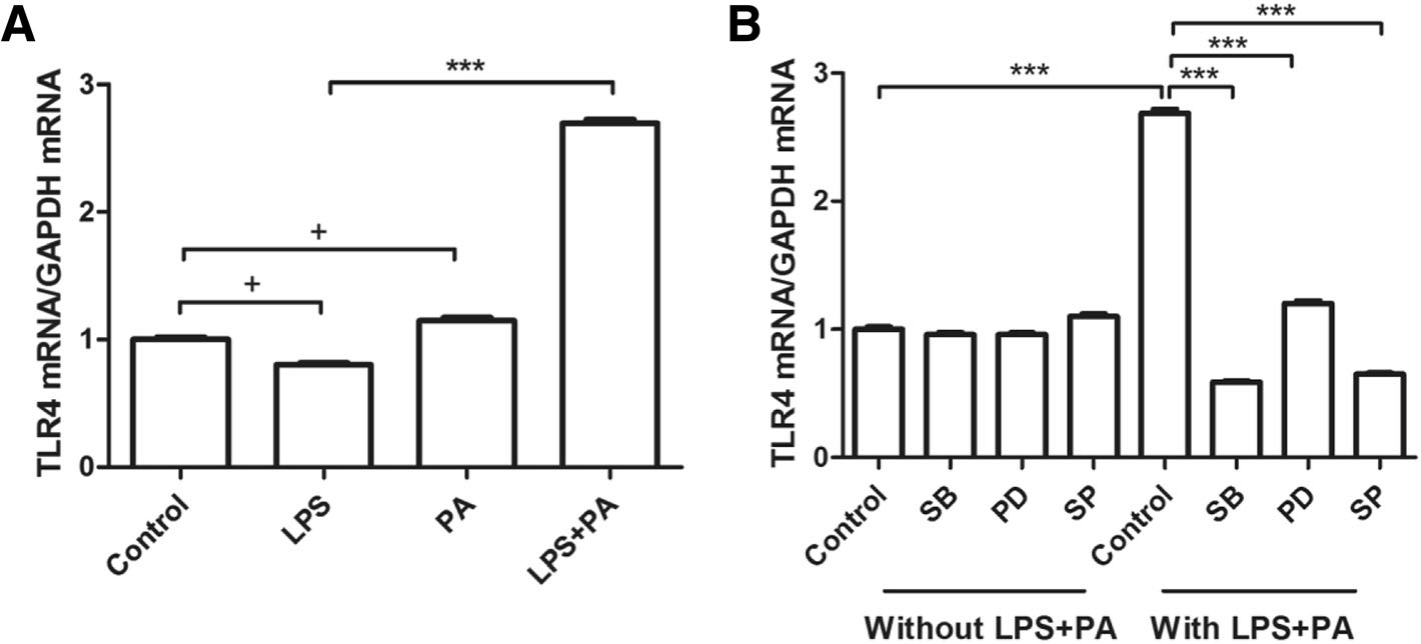
MAPK inhibitor diminished TLR4 upregulation induced by LPS + PA. **a**. RAW264.7 cells were treated with LPS 1 ng/ml, PA 100 μM, or LPS 1 ng/ml plus PA 100 μM for 24 h, then TLR4 mRNA in cells was quantified using real-time PCR. The data from three independent experiments are represented as mean ± SD. **b**. RAW264.7 cells were pretreated with 10 μM SB, 2.5 μM PD, or 2.5 μM SP for 30 min, followed by LPS plus PA treatment for 24 h, then TLR4 expression was quantified by real-time PCR. The data from three experiments were presented. ^***^ *P <* 0.01; ^+^ *P >* 0.05

The data above showed that the MAPK pathway was probably involved in MCP-1 upregulation induced by PA combined with LPS, and it is well known that LPS works via TLR4 signaling, so TLR4 mRNA level was measured in RAW264.7 cells treated with LPS plus PA treatment in the presence of MAPK inhibitors. Figure 5 showed that the elevation of TLR4 mRNA expression induced by LPS combined with PA was diminished by the inhibitors of MAPK signaling pathway such as SB, PD, and SP, indicating MCP-1 generation under LPS plus PA treatment might be via MAPK-meditated TLR4 signaling pathway.

## Discussion

This study showed that LPS could increase MCP-1 transcription as well as secretion in RAW264.7, and PA amplified this effect obviously. Meanwhile, LPS plus PA induced TLR4 mRNA expression and TLR4 knockdown inhibited MCP-1 secretion induced by LPS plus PA. Moreover, not NF-κB inhibitor but inhibitors of c-Jun NH2-terminal kinase (JNK), extracellular signal-regulated kinase (ERK), and p38 MAPK were found to block MCP-1 generation stimulated by LPS combination with PA.

Circulating MCP-1 is found to be significantly increased in patients with type II diabetes [16–18], this study confirmed macrophage secreted MCP-1 under LPS plus PA treatment, so increased MCP-1 in diabetes with obesity is at least partly from macrophage secretion. MCP-1 appears higher in the visceral and subcutaneous adipose tissues of obese patients compared to lean controls [10], and insulin induces mRNA expression and secretion of MCP-1 [19], therefore, increased MCP-1 in diabetes might be due to adipose tissues secretion.

PCR array data in our previous study revealed that PA could distinctly amplified LPS-stimulated MCP-1 mRNA expression [14]. Consistent with this report, data in the present study showed that PA could augment low-level LPS-induced MCP-1 secretion by 2.6-fold and mRNA expression by 3-fold in RAW264.7 cells. It is well known that MCP-1 mediated its effects through its receptor CCR2 [20], but the current study showed that MCP-1 generation was increased in RAW264.7 cells under PA and LPS treatment, while increased CCR2 expression was not observed, the reason might be that CCR2 expression is relatively restricted to some certain types of cells [20]. Kinetics study of mRNA expression and protein secretion in MCP-1 revealed that the synergistic increase of MCP-1 induced by LPS plus PA might be due to MCP-1 transcription, actinomycin D experiment further confirmed this hypothesis.

As a member of TLR family, TLR4 is recognized and activated by LPS, it is described to be involved in inflammatory response [21]. Together with MD-2 and CD14, TLR4 takes part in the signal transduction events initiated by LPS [22]. The current study showed that PA has the potential to work synergistically with LPS leading to TLR4 signaling activation in RAW264.7, PA plus LPS-stimulated MCP-1 secretion was due to transcriptional regulation of TLR4. In general, TLR4 acts via MyD88-dependent pathway and MyD88-independent pathway to regulate early NF-κB activation or lead to the activation of MAPK cascades to product proinflammatory cytokines [23, 24]. Thus we investigated whether NF-κB or MAPK-meditated TLR4 signaling pathway is involved in the increased generation of MCP-1 induced by PA plus LPS.

LPS could increase MCP-1 transcription by binding of NF-κB dimers to two distal NF-κB binding sites [25], the constitutive activation of NF-κB may play a role in the high level of MCP-1 production. However, the current study showed that the NF-κB inhibitor Bay-117,085 didn’t inhibited MCP-1 production induced by LPS alone or LPS combination with PA, indicating that PA augmented LPS-induced MCP-1 secretion not via NF-κB signaling pathway.

Mitogen activated protein kinases (MAPKs) are classic inflammation related signals, and JNK signaling pathway is involved in LPS-induced inflammation [26]. In this study, MCP-1 generation induced by LPS combined with PA was diminished by the inhibitors of MAPK signaling pathway such as SB, PD, and SP, indicating p38/JNK/ERK pathways were involved in MCP-1 secretion induced by LPS combination with PA. In addition, these MAPK inhibitors also abrogated the elevation of TLR4 mRNA expression induced by LPS combined with PA. Therefore, these data revealed that MCP-1 generation under LPS plus PA treatment might be via MAPK-meditated TLR4 signaling pathway.

## Conclusion

LPS and PA synergistically induced MCP-1 secretion in RAW264.7 macrophage cells, in which MCP-1 transcription mediated by MAPK/TLR4 signaling pathways was involved. Combined treatment of PA and LPS in RAW264.7 cells mimics the situation of diabetes with obesity that has higher level of PA and LPS, MAPK/TLR4/ MCP-1 might be potential therapeutic targets for diabetes with obesity.

## Abbreviations

CCK8: Cell Counting Kit-8
ELISA: Enzyme-linked immunosorbent assay
ERK: Extracellular signal-regulated kinase
FA: Fatty acid
JNK: c-Jun NH2-terminal kinase
LPS: Lipopolysaccharide
MAPK: Mitogen-activated protein kinase
MCP-1: Monocyte chemoattractant protein-1
PA: Palmitic acid
TLR4: Toll like receptor
